# Understanding the Impact of Salvage Radiation on the Long‐Term Natural History of Biochemically Recurrent Prostate Cancer After Radical Prostatectomy

**DOI:** 10.1002/cam4.70988

**Published:** 2025-06-26

**Authors:** Spyridon P. Basourakos, Stephen A. Boorjian, Phillip J. Schulte, Grant Henning, Jamie T. O'Byrne, Matthew K. Tollefson, Igor Frank, Abhinav Khanna, Ryan M. Phillips, Bradley J. Stish, R. Jeffrey Karnes, Vidit Sharma

**Affiliations:** ^1^ Department of Urology Mayo Clinic Rochester Minnesota USA; ^2^ Department of Health Sciences Research Mayo Clinic Rochester Minnesota USA; ^3^ Department of Radiation Oncology Mayo Clinic Rochester Minnesota USA

**Keywords:** biochemical recurrence, prostate cancer, salvage radiation therapy

## Abstract

**Purpose:**

The natural history of biochemical recurrence (BCR) after radical prostatectomy (RP) remains understudied, with limited long‐term data from large cohorts inclusive of both salvage radiotherapy (SRT)‐treated and untreated patients. Herein, we sought to evaluate the outcomes of patients with BCR and the impact of SRT on disease progression.

**Materials and Methods:**

Patients undergoing RP who developed BCR (PSA ≥ 0.20 ng/mL) were included. Patients with BCR treated with SRT were compared to untreated patients using risk‐set matching with time‐dependent propensity scores. The primary outcome was metastases, analyzed using Kaplan–Meier and Cox models. The number needed to treat (NNT) with SRT to prevent progression was derived at 5 and 15 years.

**Results:**

Among 6881 patients with BCR, 2109 received SRT. At a median follow‐up of 10.2 years, 1147 patients developed metastases. The median PSA at the time of SRT was 0.50 ng/mL. After 1:1 propensity score matching (2109 patients per cohort), SRT significantly reduced the risk of metastases at 5 (12.7% vs. 19.3%, *p* < 0.0001) and 15 years (28.6% vs. 31.5%, *p* < 0.001). On multivariable analysis, SRT independently reduced metastasis risk (HR 0.75, 95% CI 0.63–0.90, *p* = 0.002), translating to NNT of 23 and 15 at 5 and 15 years, respectively. Interaction analyses between SRT and nodal status (*p* = 0.04) showed greater metastasis risk reduction in pN+ (HR 0.41, 95% CI 0.22–0.77, *p* = 0.005) compared to pN− disease (HR 0.81, 95% CI 0.67–0.97, *p* = 0.02).

**Conclusions:**

Most patients with BCR post‐RP do not progress to metastasis. For those who do progress, SRT inarguably improves the oncologic outcomes in the BCR setting. However, careful patient selection and shared decision making should be encouraged in order to limit overtreatment and side effects.

## 1 Introduction

Up to one third of men undergoing radical prostatectomy (RP) have been reported to experience biochemical recurrence (BCR) within 10 years [1, 2, 3, 4]. Although BCR serves as a precursor to systemic disease, its natural history is heterogeneous and typically quite prolonged, which creates challenges for management and underscores the importance of considering the toxicity and costs of secondary treatments, as well as patients' competing risks of mortality [2, 5]. Contemporary guidelines which advocate for “early” salvage radiation therapy (SRT) provide supporting data that the effectiveness of SRT decreases as PSA levels rise [6]. Indeed, reports from our center [7] and others [8] demonstrate improved oncologic outcomes with SRT delivered when postoperative PSA is ≤ 0.50 ng/mL compared to higher levels. Moreover, Tilki et al. [9] recently demonstrated that initiating SRT at PSA < 0.25 ng/mL is associated with a lower risk of overall mortality (OM).

However, a critical limitation of most studies to date examining the outcomes of SRT at various postoperative PSA levels is the absence of a comparator, or “control,” population of patients with BCR at the same PSA levels who are not treated with SRT. That is, reported SRT studies have primarily consisted exclusively of SRT‐treated patients and compared the outcomes following SRT delivered to patients with a lower versus a higher level of PSA. Notably, we previously documented that 40% of patients who experience a PSA rise to 0.20 ng/mL post‐RP and remain untreated do not experience a subsequent continued PSA increase, and in fact only 10% of patients with a PSA of 0.20 ng/mL who do not receive SRT develop metastasis by 10 years [1, 10]. Thus, we believe that including an untreated comparator cohort is essential to determining the impact of SRT on the natural history of BCR following RP.

To our knowledge, only two retrospective series to date have examined the impact of SRT on metastases and mortality among men with BCR, including a cohort of patients with BCR who did not receive SRT [11, 12]. However, these studies were limited by relatively small sample sizes and the use of historic cohorts [11, 12]. Therefore, we sought to evaluate the long‐term natural history of patients with BCR after RP and quantify the oncologic benefit of SRT in a large cohort with extended follow‐up. Specifically, using propensity score matching (PSM), we compared metastasis, prostate cancer‐specific mortality (PCSM), and OM among patients with BCR managed with or without SRT. We then performed interaction analyses to identify subsets of patients most likely to benefit from SRT.

## 2 Materials and Methods

### 2.1 Cohort Description

**FIGURE 1:**
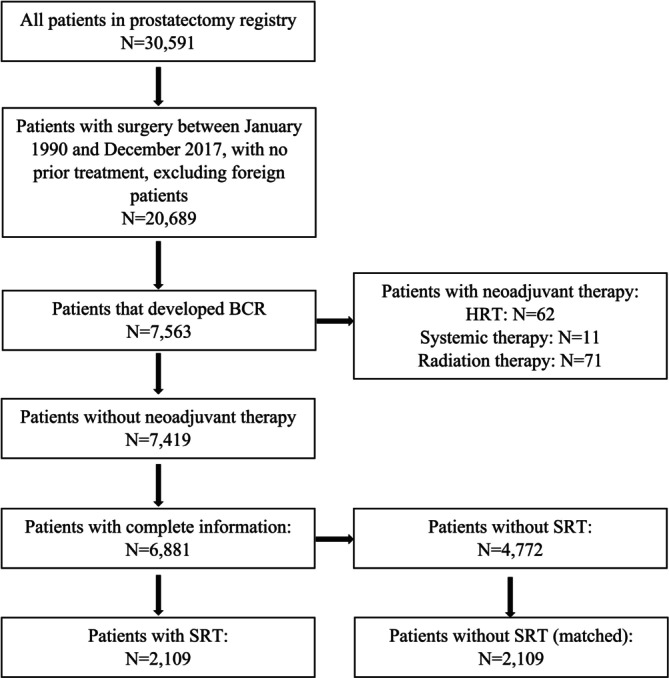
Consort diagram of BCR cohort selection.

Following Mayo Clinic Institutional Review Board approval (No. 23‐008224), we analyzed consecutive patients who underwent RP for localized prostate cancer at Mayo Clinic from January 1990 to December 2017 and subsequently developed BCR. BCR was defined as two consecutive post‐RP PSA values > 0.20 ng/mL. We excluded men who received neoadjuvant hormone therapy (*N* = 62), adjuvant systemic therapy (*N* = 11), or adjuvant radiation therapy (*N* = 71) (Figure 1). RP was performed using standardized open or minimally invasive techniques. The extent of pelvic lymph node dissection varied by surgeon and over the study period. Patients with positive lymph nodes post‐RP were included. The decision to pursue SRT was at the discretion of the patient and the treating urologist and radiation oncologist.

### 2.2 Radiation Therapy

The radiographic evaluation prior to SRT, the use of concurrent androgen deprivation therapy (ADT) and the specific SRT treatment plan, target localization, and delivery methods were determined by the treating radiation oncologist. Post‐SRT monitoring primarily involved regular PSA measurements and clinical examinations, with additional tests as indicated. Salvage ADT for rising PSA or clinical progression following SRT was also initiated at the treating clinician's discretion. We did not adjust for salvage ADT as this treatment has shown no consistent impact on outcomes [13, 14], and indeed the AUA Advanced Prostate Cancer Guidelines do not endorse routine ADT for BCR [15].

### 2.3 Outcomes

The primary outcome of interest was metastases. The diagnosis of metastases was achieved using various imaging modalities: computed tomography (CT), bone scan, magnetic resonance imaging (MRI), 11C‐choline PET/CT, 18F‐choline PET/CT, and Ga‐68‐PSMA PET/CT. These assessments were supplemented with pathologic confirmation as deemed appropriate by the treating physician. Secondary outcomes included PCSM and OM. Patients' vital status was determined annually from either death certificates or direct communication with physicians.

### 2.4 Descriptive Statistics

Descriptive statistics were performed to compare clinical features between SRT and matched surveillance groups, summarized by absolute standardized difference (ASD). ASD is defined as the difference in means (or proportions) divided by the standard deviation and is a common approach for evaluating whether matching has satisfied balance assumptions. ASD < 0.2 is interpreted as well balanced in the matched cohort. Statistical analyses were performed using SAS version 9.1.3 statistical software (SAS Institute, Cary, NC, USA), and all tests were two‐sided with a *p* ≤ 0.05 considered significant.

### 2.5 Time‐Dependent Propensity Score Matching

We conducted risk set matching between patients who did and did not receive SRT for BCR using a time‐dependent propensity score. The hazard for SRT treatment over time post‐BCR was modeled using a Cox proportional hazards model. Covariates in the Cox regression for SRT treatment were selected a priori based on suspected confounders, including time‐independent covariates at surgery or at BCR (time zero): age at BCR, year of surgery, time from surgery to BCR, Gleason grade group, T stage, N stage, and margin status at RP [8, 16]. We also included time‐dependent covariates ascertained after BCR: PSA value, logarithm of PSA, highest postoperative PSA, and PSA count since surgery. The inclusion of multiple functions of PSA allows for a flexible nonlinear functional form of the relationship (including PSA and log PSA in the model). Furthermore, we did not include adjustment for PSA doubling time (PSADT) in the matching process because the date to start the time frame for the calculation of PSADT is not known for the non‐SRT patients until they have been matched with an SRT patient. However, we performed a subset analysis to include only patients with PSADT information, and the PSADT is controlled for in the models. Time‐dependent covariates were updated at each new PSA report in the registry record for the patient. The model outputs a propensity to be treated with SRT for every patient at every time point between BCR and observed SRT or loss to follow‐up (including death). Further details regarding PSM are provided in Supplementary Methods.

The association between surveillance and SRT with metastasis risk was assessed using a Cox proportional hazards model, with time zero defined as the time of SRT or the matched equivalent for surveillance patients. Cause‐specific hazards models were used to account for competing mortality risks. PCSM was similarly analyzed. The number needed to treat (NNT) with SRT was calculated using regression estimated absolute risk reduction according to Altman's method [17].

## 3 Results

### 3.1 Cohort Characteristics

Among 20,689 patients who underwent RP for localized prostate cancer during the study period, 7563 developed BCR and 6881 had available data for analysis (Figure 1). The median follow‐up after BCR was 10.2 years (IQR 5.0, 16.3). During this period, 1147 (16.7%) patients with BCR developed metastases and 1761 died (507 from prostate cancer). In matched patients with BCR who did not receive SRT, the 15‐year risks of metastases, PCSM, and OM were 30%, 18%, and 45%, respectively.

### 3.2 Association of SRT With Metastasis Among Men With BCR

Overall, 2109 patients who developed BCR received SRT. Patients with BCR who were treated with SRT were younger (64.6 vs. 68.5, *p* < 0.001), less frequently had lymph node–positive disease (3.5% vs. 6.6%, *p* < 0.001), and were more likely to have positive surgical margins (45.9% vs. 37.5%, *p* = 0.15) than untreated patients (Table 1). After PSM, the ASD between the SRT and surveillance cohorts was negligible.

**TABLE 1:** Clinicopathologic characteristics of patients with BCR, stratified by receipt of SRT, before and after PSM.

	Entire cohort	Prior to propensity score matching			After propensity score matching			
	*N* = 6881	Surveillance (*N* = 4772)	SRT (*N* = 2109)	Absolute standardized differences*	Surveillance (*N* = 2109)	SRT (*N* = 2109)	Absolute standardized differences*	*p*
Age at BCR Median (IQR)	67.7 (62.0, 72.7)	68.9 (63.3, 73.8)	65.1 (59.6, 69.7)	0.263	66.5 (60.3, 70.7)	65.1 (59.6, 69.7)	0.120	< 0.001^a^
Year of surgery, *N* (%)				0.648			0.034	< 0.001^b^
1990–1994	2003 (29.1)	1617 (33.9)	386 (18.3)		429 (20.3)	386 (18.3)		
1995–1999	1652 (24)	1219 (25.5)	433 (20.5)		435 (20.6)	433 (20.5)		
2000–2004	1145 (16.6)	710 (14.9)	435 (20.6)		328 (15.6)	435 (20.6)		
2005–2009	1117 (16.2)	657 (13.8)	460 (21.8)		442 (21.0)	460 (21.8)		
2010–2014	646 (9.4)	382 (8)	264 (12.5)		298 (14.1)	264 (12.5)		
2015–2017	318 (4.6)	187 (3.9)	131 (6.2)		177 (8.4)	131 (6.2)		
Pathology Gleason score, *N* (%)				0.525			0.124	< 0.001^b^
6	2973 (43.2)	2313 (48.5)	660 (31.3)		576 (27.3)	660 (31.3)		
7	3077 (44.7)	1920 (40.2)	1157 (54.9)		1166 (55.3)	1157 (54.9)		
≥ 8	831 (12.1)	539 (11.3)	292 (13.8)		367 (17.4)	292 (13.8)		
Pathology T stage, *N* (%)				0.145			0.042	0.113^b^
T2	4522 (65.7)	3209 (67.2)	1313 (62.3)		1261 (59.8)	1313 (62.2)		
T3a	1294 (18.8)	852 (17.9)	442 (21.0)		444 (21.1)	442 (21.0)		
T3b/T4	1065 (15.5)	711 (14.9)	354 (16.8)		404 (19.1)	354 (16.8)		
Positive surgical margin, *N* (%)	2756 (40.1)	1788 (37.5)	968 (45.9)	0.146	985 (46.7)	968 (45.9)	0.016	0.600^b^
Positive nodes, *N* (%)	386 (5.6)	313 (6.6)	73 (3.5)	0.063	113 (5.4)	73 (3.5)	0.092	0.003^b^
Years from surgery to BCR, median (IQR)	2.5 (0.9, 5.5)	2.9 (1.0, 6.5)	1.7 (0.7, 3.6)	0.246	1.7 (0.6, 3.7)	1.7 (0.7, 3.6)	0.001	0.304^a^
PSA value at the time of matching, median (IQR)					0.5 (0.3, 1.3)	0.5 (0.3, 1.1)	0.020	0.766^a^
Number of PSA checks between BCR and SRT or matching date, median (IQR)					3.0 (2.0, 6.0)	3.0 (2.0, 6.0)	0.020	0.163^a^

* The absolute standardized differences are a metric that attempts to evaluate the assumption that the propensity score is providing adequate balance between groups. Due to our large *N*, many of these *p* values would be taken to indicate significant differences between the two groups despite there being a lot of overlap in the IQRs and minimal absolute standardized differences, indicating similar group distributions of matched characteristics.

^a^ Kruskal–Wallis *p* value.

^b^ Chi‐square *p* value.

**FIGURE 2:**
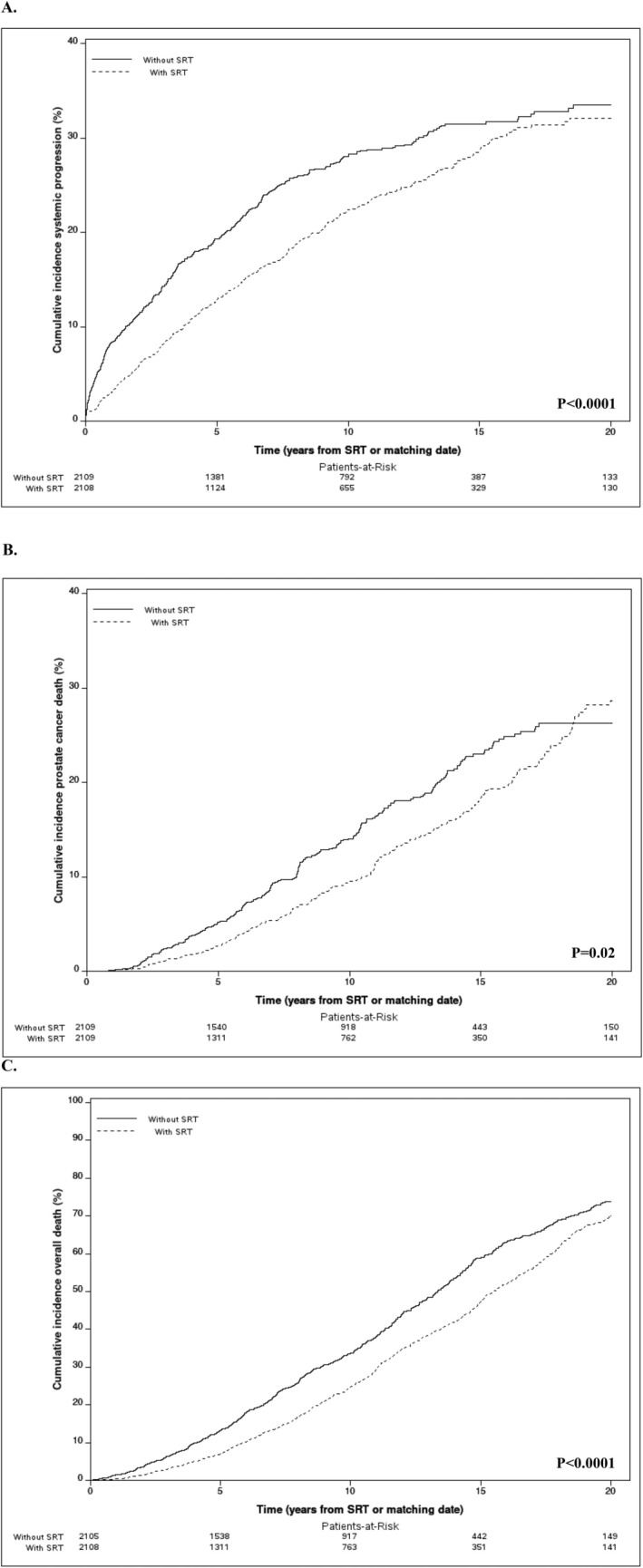
Cumulative incidence of (A) systemic progression, (B) prostate cancer‐specific mortality, and (C) overall mortality among 1:1 propensity score‐matched patients with BCR post‐RP, stratified by receipt of SRT versus no SRT.

Among PSM patients with BCR, those who received SRT had significantly lower risks of metastasis at 5 (12.7% vs. 19.3%, *p* < 0.0001) and 15 years (28.6% vs. 31.5%, *p* < 0.0001) compared to patients not treated with SRT (Figure 2A). Similar findings were noted in the subset of patients with sufficient PSADT data (Supplementary Table and Supplementary Figure). Receipt of SRT remained associated with a decreased risk of metastases on multivariable analysis as well (HR 0.75, 95% CI 0.63–0.90, *p* = 0.002). These data translate to a NNT with SRT for BCR of 23 (95% CI 17–41) and 15 (95% CI 11–27) to prevent one metastasis at 5 and 15 years, respectively (Table 2).

**TABLE 2:** Hazard ratios of SRT after matching by propensity scores and number needed to treat for systemic progression, prostate cancer specific mortality, and overall mortality.

Disease outcome	Hazard ratio (95% CI)	*p*	Number needed to treat* (95% CI)	
			5 years	15 years
Systemic progression	0.75 (0.63–0.90)	**0.002**	23 (17–41)	15 (11–27)
Prostate cancer mortality	0.81 (0.64–1.03)	0.086	103 (62–494)	26 (15–124)
Overall mortality	0.79 (0.70–0.89)	**0.0001**	38 (29–62)	12 (9–20)

* NNT calculated using regression‐based absolute differences in event‐free survival between SRT and observation.

### 3.3 Association of SRT With PCSM and OM Among Men With BCR

Within the PSM cohort, we noted that PCSM at 5 years was 2.6% versus 5.2% in patients who did versus did not receive SRT, and 18.3% versus 23.0% at 15 years (*p* = 0.02) (Figure 2B). The corresponding risks of OM were 7.0% versus 13.3% at 5 years and 47.2% versus 58.9% at 15 years (*p* < 0.0001), respectively (Figure 2C). On multivariable analysis, SRT was independently associated with reduced OM (HR 0.79, 95% CI 0.70–0.89; *p* = 0.0001), but did not reach statistical significance for PCSM (HR 0.81, 95% CI 0.64–1.03; *p* = 0.09).

### 3.4 Interaction Analysis for Factors Associated With the Magnitude of Benefit From SRT

To evaluate the potential relationship between various clinicopathologic factors and the efficacy of SRT, which might then facilitate a more individualized approach to BCR management, we conducted interaction analyses evaluating nodal status, surgical margin status, pathologic T stage, and PSA levels prior to SRT (Table 3). Specifically, we found no interaction (*p* value for interaction > 0.10) with respect to the association of SRT with metastasis and margin status, pathologic T stage, and pathologic Gleason score. On the other hand, a significant interaction was identified between SRT and nodal status (*p* = 0.04), such that SRT was associated with a greater metastasis risk reduction among pN+ SRT‐treated patients (HR 0.41, 95% CI 0.22–0.77, *p* = 0.005) than pN− patients (HR 0.81, 95% CI 0.67–0.97, *p* = 0.02). However, pN status did not significantly interact with the effect size of SRT for the outcome of PCSM (*p* = 0.33) or OM (*p* = 0.16). Importantly, pre‐SRT PSA (≤ 0.40 vs. > 0.40 ng/mL) was the only variable modifying the effect of SRT on metastasis (*p* = 0.02), PCSM (*p* = 0.003), and OM (*p* = 0.009). Furthermore, for the cohort of patients for whom PSADT values were available, the effect size of SRT on metastasis (*p* = 0.88), PCSM (*p* = 0.42), and OM (*p* = 0.93) did not vary significantly across PSADT subsets (greater than or less than 1 year) (Table 3).

**TABLE 3:** Comparisons of systemic progression, prostate‐cancer‐specific mortality, and overall mortality between surveillance and SRT matched patients, including interaction analysis for nodal status, margin status, pathologic T stage, composite pT stage and margin status, last PSA prior to SRT, pathologic Gleason score, and PSA doubling time.

		Systemic progression		Prostate cancer‐specific mortality		Overall mortality	
Variable of interest		HR (95% CI)	*p*	HR (95% CI)	*p*	HR (95% CI)	*p*
Nodal status	N0	0.81 (0.67–0.97)	0.02	0.85 (0.66–1.10)	0.22	0.80 (0.71–0.94)	0.0006
	N1	0.41 (0.22–0.77)	0.005	0.60 (0.31–1.16)	0.13	0.52 (0.29–0.94)	0.03
	Interaction	—	0.04	—	0.33	—	0.16
Margin status	Negative margin	0.73 (0.58–0.92)	0.01	1.08 (0.75–1.54)	0.68	0.90 (0.75–1.08)	0.25
	Positive margin	0.78 (0.60–1.02)	0.07	0.65 (0.47–0.90)	0.009	0.70 (0.60–0.83)	< 0.0001
	Interaction	—	0.68	—	0.04	—	0.047
Pathologic T stage	T2	0.83 (0.63–1.10)	0.19	1.20 (0.80–1.79)	0.38	0.85 (0.72–0.99)	0.04
	T3a	0.79 (0.55–1.13)	0.19	0.69 (0.42–1.15)	0.15	0.72 (0.54–0.95)	0.02
	T3b/T4	0.72 (0.54–0.96)	0.03	0.74 (0.51–1.06)	0.10	0.78 (0.60–1.02)	0.07
	Interaction	—	0.79	—	0.13	—	0.58
Composite pT and margin	pT2 and R0	0.84 (0.60–1.18)	0.31	1.70 (0.97–2.96)	0.06	1.00 (0.81–1.24)	0.98
	pT3/4 or R1	0.71 (0.58–0.87)	0.001	0.66 (0.50–0.86)	0.002	0.71 (0.61–0.82)	< 0.0001
	Interaction	—	0.41	—	0.003		0.009
Last PSA prior to SRT	≤ 0.4 ng/mL	0.99 (0.73–1.35)	0.95	1.29 (0.79–2.12)	0.31	0.99 (0.81–1.21)	0.92
	> 0.4 ng/ml	0.66 (0.54–0.80)	< 0.0001	0.67 (0.51–0.87)	0.003	0.68 (0.59–0.79)	< 0.0001
	Interaction	—	0.02	—	0.02	—	0.003
Pathologic Gleason score	6	1.06 (0.68–1.65)	0.80	1.30 (0.73–2.31)	0.37	0.94 (0.78–1.14)	0.53
	7	0.75 (0.60–0.94)	0.01	0.86 (0.62–1.18)	0.35	0.79 (0.66–0.94)	0.009
	≥ 8	0.75 (0.53–1.08)	0.12	0.67 (0.44–1.09)	0.11	0.62 (0.45–0.87)	0.005
	Interaction	—	0.38	—	0.23	—	0.08
PSA doubling time*	≥ 1 year	0.77 (0.50–1.19)	0.24	1.01 (0.51–1.99)	0.97	0.84 (0.64–1.11)	0.21
	< 1 year	0.74 (0.54–1.01)	0.054	0.73 (0.47–1.12)	0.15	0.82 (0.65–1.04)	0.10
	Interaction	—	0.88	—	0.42	—	0.93

* Model is subset to only patients with PSA doubling time information.

## 4 Discussion

Herein, we evaluated what is to our knowledge the largest reported cohort of patients with BCR to determine long‐term oncologic outcomes. We determined that approximately 70% of patients with BCR who did not receive SRT remained metastasis‐free at 15 years, underscoring the relatively indolent natural history of most patients with a detectable PSA after surgery. Using a time‐dependent PSM approach, we quantified the benefit of SRT in lowering the risks of metastasis, PCSM, and OM. SRT was associated with absolute reductions in metastasis risk of 6.6% at 5 years and 2.9% at 15 years, translating to NNT of 23 and 15 patients, respectively, at these time points. Again, such data emphasize the importance of careful patient selection for secondary treatments to avoid exposing those patients not likely to experience disease progression to the costs and potential toxicities of secondary therapy. Moreover, our interaction analyses suggest that certain subgroups—particularly those with lymph node–positive disease—may derive the greatest benefit from SRT.

Our study provides a contemporary perspective on the long‐term natural history of untreated BCR [1, 18]. Specifically, by demonstrating that many patients with guideline‐defined BCR [19] will never progress clinically, our findings support the value of a risk‐stratified approach to BCR. Indeed, several risk models have been proposed for clinical use [3, 20, 21, 22]. Such an individualized management strategy optimizes the risk/benefit/cost balance of SRT, sparing patients with low‐risk BCR from unnecessary additional treatment. While most patients who receive post‐RP radiation report favorable long‐term quality of life [23], there is a risk for treatment‐related complications requiring urologic or gastrointestinal intervention, as well as increased risk for hospitalization and secondary malignancy [24].

In addition to large cohort size and long‐term follow‐up, our study is noteworthy for including patients with BCR who did and did not receive SRT. Two prior retrospective series have compared outcomes of patients with BCR managed with SRT versus surveillance. Trock et al. [11] analyzed 635 patients with BCR (238 received SRT) treated from 1980 to 2004 and reported that SRT significantly improved PCSM relative to surveillance. In a second study, Tilki et al. [12] examined 874 PSM patients with BCR managed with SRT versus surveillance and demonstrated that SRT was associated with a 9% reduction in metastasis. However, Tilki et al. did not analyze whether specific subsets of patients particularly benefited from SRT, and in addition, the propensity score used there solely considered surgical and pathologic characteristics, omitting post‐RP PSA values. Consequently, that model did not account for elapsed times since BCR, potentially introducing immortal time bias, as patients receiving SRT are assumed to survive to a certain point post‐BCR to receive treatment, a condition not applicable to their untreated counterparts. Our study significantly improves on these earlier works [11, 12] by including over 4100 PSM patients and by using a time‐dependent propensity score, enabling outcome evaluation from the initiation of SRT or its matched equivalent (such as time from RP and post‐RP PSA), effectively mitigating immortal time bias.

Our comparative analysis enabled us to determine the NNT with SRT, highlighting the potential overtreatment of frequently indolent disease and further emphasizing the importance of risk stratification and shared decision making in the management of BCR [6, 22]. In fact, we would submit that our findings establish a strong rationale for a trial comparing early SRT to surveillance among patients with BCR after RP. Additionally, our demonstration of an interaction between PSA at the time of SRT and subsequent oncologic outcomes underscores the need to consider each patient's clinicopathologic risk factors when deciding whether to initiate treatment at a postoperative detectable PSA. Not all patients with the same PSA value are at comparable risk of progression, nor are they likely to derive equivalent benefit from secondary local therapy. Meanwhile, our interaction analysis suggests that SRT may be particularly beneficial in decreasing the risk of metastases among patients with pN+ disease, an especially high‐risk cohort for whom the optimal management after surgery remains incompletely defined.

We acknowledge that the metastases‐free survival outcomes reported in our study differ from those observed in prior institutional series of patients with BCR following RP. Importantly, in a prior series from our institution, Boorjian et al., utilizing data between 1990 and 2006, similarly reported metastasis in approximately 20% of patients with BCR after RP at 10 years and 24% at 15 years [1]. Meanwhile, Pound et al. [4] reported a 15‐year metastasis‐free survival of just over 25% after BCR, and Freedland et al. [5] observed a 15‐year cancer‐specific survival of 53% among 379 men with post‐RP BCR, both analyzing cohorts from Johns Hopkins. We would submit that the reasons for the disparate reported outcomes between these different institutional series are likely manifold. For one, differences in cohort clinicopathologic characteristics likely contributed to the observed differences in outcomes. For example, in a separate series from Hopkins, Trock et al. [11] studied a cohort where 75%–80% of patients had Grade 7 or higher disease, compared to 56% in our study. Additionally, their cohort included less than 20% with pT2 disease, whereas our study included 65% with pT2. These differences suggest that the Hopkins cohort may have comprised patients with more aggressive or advanced disease, potentially explaining the higher metastasis rates reported from those studies. In addition, reported metastasis‐free survival rates may be influenced by differences in the timing of salvage therapy delivery, details of salvage therapy delivery, follow‐up duration, as well as differential use of the imaging modalities which have evolved over time (e.g., PSMA PET scan).

We recognize that our study is limited by its retrospective design, spanning multiple years at a single institution. Indeed, despite employing matched pair analysis, we cannot preclude the presence of unknown confounders that may have impacted decisions regarding SRT. We acknowledge that our study lacks specific details regarding SRT, such as radiation dose and concurrent use of ADT, largely due to the extended study period over which practice patterns evolved. Additionally, we were unable to assess the impact of salvage ADT on outcomes. We did not include PSADT as a matching variable in our main analysis to avoid significantly reducing the pool of eligible participants due to difficulties in matching on this constantly varying parameter [25], however, our subset analysis for patients with available PSADT values did not significantly change our results. Moreover, the application of Altman's method to estimate the NNT introduces inherent variability, as this approach assumes a fixed survival rate for controls at the selected time “*t*.” Furthermore, in evolving imaging modalities the use of postoperative imaging is not captured as a variable in our registry and given the prolonged time frame over which patients were included and the large size of the cohort here, we are not able to comprehensively and reliable obtain these data for analysis. Given the prolonged time frame over which patients were included here for analysis, we acknowledge the evolution in staging modalities which has occurred (e.g., bone scan to PSMA PET scan) over the study period may have led to earlier radiographic detection of disease [26, 27]. We did not separately analyze the outcomes following SRT for patients with BCR and radiographic evidence of local recurrence, as we have previously reported [28]. Collectively, these confounders would be best addressed with a prospective randomized trial to define the impact of SRT on the natural history of BCR and properly quantitate the benefit of such additional local therapy in preventing clinical progression.

## 5 Conclusions

Most patients with BCR after RP do not develop metastases on long‐term follow‐up. Using a time‐varying PSM algorithm with over 4100 patients, we determined that SRT was associated with an approximately 6.6% and 2.9% reduction in metastasis at 5 and 15 years, respectively. These findings suggest that SRT contributes to a meaningful reduction in metastasis in the post‐BCR setting, while also underscoring the importance of a risk‐stratified approach to BCR management. Such an approach seeks to identify patients most likely to benefit from SRT, while carefully considering potential toxicity and costs. These results offer valuable insights for patient counseling and support shared decision making regarding the management of postoperative BCR. Further randomized trials are warranted to refine treatment strategies in this setting.

## Author Contributions

**Spyridon P. Basourakos:** conceptualization (equal), formal analysis (equal), investigation (equal), methodology (equal), writing – original draft (equal), writing – review and editing (equal). **Stephen A. Boorjian:** conceptualization (equal), funding acquisition (equal), investigation (equal), methodology (equal), project administration (equal), resources (equal), supervision (equal), validation (equal), writing – original draft (equal), writing – review and editing (equal). **Phillip J. Schulte:** data curation (equal), formal analysis (equal), methodology (equal), software (equal), validation (equal). **Grant Henning:** investigation (equal), writing – original draft (equal), writing – review and editing (equal). **Jamie T. O'Byrne:** data curation (equal), formal analysis (equal), methodology (equal), software (equal). **Matthew K. Tollefson:** conceptualization (equal), investigation (equal), methodology (equal), writing – review and editing (equal). **Igor Frank:** methodology (equal), supervision (equal), writing – review and editing (equal). **Abhinav Khanna:** investigation (equal), supervision (equal), writing – review and editing (equal). **Ryan M. Phillips:** investigation (equal), supervision (equal), writing – review and editing (equal). **Bradley J. Stish:** investigation (equal), supervision (equal), writing – review and editing (equal). **R. Jeffrey Karnes:** conceptualization (equal), supervision (equal), writing – review and editing (equal). **Vidit Sharma:** conceptualization (equal), formal analysis (equal), funding acquisition (equal), investigation (equal), methodology (equal), project administration (equal), resources (equal), supervision (equal), writing – review and editing (equal).

## Consent

Written informed consent was obtained by the study participants.

## Conflicts of Interest

Vidit Sharma owns personal stock worth < $10,000 in ImmunityBio and Macrogenics. Stephen A. Boorjian is a consultant for Ferring, ArTara, Prokarium, and Johnson & Johnson.

## Supporting information


**Data S1.** Supporting Information.

## Data Availability

Data supporting this study are available from the corresponding author upon reasonable request.

## References

[1] S. A. Boorjian , R. H. Thompson , M. K. Tollefson , et al., “Long‐Term Risk of Clinical Progression After Biochemical Recurrence Following Radical Prostatectomy: The Impact of Time From Surgery to Recurrence,” European Urology 59 (2011): 893–899. 21388736 10.1016/j.eururo.2011.02.026

[2] J. F. Ward , M. L. Blute , J. Slezak , et al., “The Long‐Term Clinical Impact of Biochemical Recurrence of Prostate Cancer 5 or More Years After Radical Prostatectomy,” Journal of Urology 170 (2003): 1872–1876. 14532796 10.1097/01.ju.0000091876.13656.2e

[3] J. A. Brockman , S. Alanee , A. J. Vickers , et al., “Nomogram Predicting Prostate Cancer‐Specific Mortality for Men With Biochemical Recurrence After Radical Prostatectomy,” European Urology 67 (2015): 1160–1167. 25301759 10.1016/j.eururo.2014.09.019 PMC4779062

[4] C. R. Pound , A. W. Partin , M. A. Eisenberger , D. W. Chan , J. D. Pearson , and P. C. Walsh , “Natural History of Progression After PSA Elevation Following Radical Prostatectomy,” JAMA 281 (1999): 1591–1597. 10235151 10.1001/jama.281.17.1591

[5] S. J. Freedland , E. B. Humphreys , L. A. Mangold , et al., “Risk of Prostate Cancer‐Specific Mortality Following Biochemical Recurrence After Radical Prostatectomy,” JAMA 294 (2005): 433. 16046649 10.1001/jama.294.4.433

[6] T. M. Morgan , S. A. Boorjian , M. K. Buyyounouski , et al., “Salvage Therapy for Prostate Cancer: AUA/ASTRO/SUO Guideline Part I: Introduction and Treatment Decision‐Making at the Time of Suspected Biochemical Recurrence After Radical Prostatectomy,” Journal of Urology 211 (2024): 509–517. 38421253 10.1097/JU.0000000000003892

[7] B. J. Stish , T. M. Pisansky , W. S. Harmsen , et al., “Improved Metastasis‐Free and Survival Outcomes With Early Salvage Radiotherapy in Men With Detectable Prostate‐Specific Antigen After Prostatectomy for Prostate Cancer,” Journal of Clinical Oncology 34 (2016): 3864–3871. 27480153 10.1200/JCO.2016.68.3425

[8] R. D. Tendulkar , S. Agrawal , T. Gao , et al., “Contemporary Update of a Multi‐Institutional Predictive Nomogram for Salvage Radiotherapy After Radical Prostatectomy,” Journal of Clinical Oncology 34 (2016): 3648–3654. 27528718 10.1200/JCO.2016.67.9647

[9] D. Tilki , M. H. Chen , J. Wu , et al., “Prostate‐Specific Antigen Level at the Time of Salvage Therapy After Radical Prostatectomy for Prostate Cancer and the Risk of Death,” Journal of Clinical Oncology 41 (2023): 2428–2435. 36857638 10.1200/JCO.22.02489 PMC10150889

[10] A. Toussi , S. B. Stewart‐Merrill , S. A. Boorjian , et al., “Standardizing the Definition of Biochemical Recurrence After Radical Prostatectomy‐What Prostate Specific Antigen Cut Point Best Predicts a Durable Increase and Subsequent Systemic Progression?,” Journal of Urology 195 (2016): 1754–1759. 26721226 10.1016/j.juro.2015.12.075

[11] B. J. Trock , M. Han , S. J. Freedland , et al., “Prostate Cancer‐Specific Survival Following Salvage Radiotherapy vs Observation in Men With Biochemical Recurrence After Radical Prostatectomy,” JAMA 299 (2008): 2760–2769. 18560003 10.1001/jama.299.23.2760 PMC3076799

[12] D. Tilki , F. Preisser , R. Thamm , et al., “Salvage Radiotherapy Versus Observation for Biochemical Recurrence Following Radical Prostatectomy for Prostate Cancer: A Matched Pair Analysis,” Cancers (Basel) 14 (2022): 740. 35159007 10.3390/cancers14030740 PMC8833698

[13] J. W. Moul , H. Wu , L. Sun , et al., “Early Versus Delayed Hormonal Therapy for Prostate Specific Antigen Only Recurrence of Prostate Cancer After Radical Prostatectomy,” Journal of Urology 171 (2004): 1141–1147. 14767288 10.1097/01.ju.0000113794.34810.d0

[14] X. Garcia‐Albeniz , J. M. Chan , A. Paciorek , et al., “Immediate Versus Deferred Initiation of Androgen Deprivation Therapy in Prostate Cancer Patients With PSA‐Only Relapse. An Observational Follow‐Up Study,” European Journal of Cancer 51 (2015): 817–824. 25794605 10.1016/j.ejca.2015.03.003 PMC4402138

[15] W. T. Lowrance , R. H. Breau , R. Chou , et al., “Advanced Prostate Cancer: AUA/ASTRO/SUO Guideline PART I,” Journal of Urology 205 (2021): 14–21. 32960679 10.1097/JU.0000000000001375

[16] A. J. Stephenson , P. T. Scardino , M. W. Kattan , et al., “Predicting the Outcome of Salvage Radiation Therapy for Recurrent Prostate Cancer After Radical Prostatectomy,” Journal of Clinical Oncology 25 (2007): 2035–2041. 17513807 10.1200/JCO.2006.08.9607 PMC2670394

[17] D. G. Altman , “Confidence Intervals for the Number Needed to Treat,” BMJ 317 (1998): 1309–1312. 9804726 10.1136/bmj.317.7168.1309 PMC1114210

[18] S. A. Boorjian , R. J. Karnes , P. L. Crispen , L. J. Rangel , E. J. Bergstralh , and M. L. Blute , “Radiation Therapy After Radical Prostatectomy: Impact on Metastasis and Survival,” Journal of Urology 182 (2009): 2708–2715. 19836762 10.1016/j.juro.2009.08.027

[19] I. M. Thompson , R. K. Valicenti , P. Albertsen , et al., “Adjuvant and Salvage Radiotherapy After Prostatectomy: AUA/ASTRO Guideline,” Journal of Urology 190 (2013): 441–449. 23707439 10.1016/j.juro.2013.05.032

[20] T. Van den Broeck , R. C. N. van den Bergh , N. Arfi , et al., “Prognostic Value of Biochemical Recurrence Following Treatment With Curative Intent for Prostate Cancer: A Systematic Review,” European Urology 75 (2019): 967–987. 30342843 10.1016/j.eururo.2018.10.011

[21] D. Tilki , F. Preisser , M. Graefen , H. Huland , and R. S. Pompe , “External Validation of the European Association of Urology Biochemical Recurrence Risk Groups to Predict Metastasis and Mortality After Radical Prostatectomy in a European Cohort,” European Urology 75 (2019): 896–900. 30955970 10.1016/j.eururo.2019.03.016

[22] T. M. Morgan , S. A. Boorjian , M. K. Buyyounouski , et al., “Salvage Therapy for Prostate Cancer: AUA/ASTRO/SUO Guideline Part II: Treatment Delivery for Non‐Metastatic Biochemical Recurrence After Primary Radical Prostatectomy,” Journal of Urology 211 (2024): 518–525. 38421243 10.1097/JU.0000000000003891

[23] A. S. Akthar , C. Liao , S. E. Eggener , and S. L. Liauw , “Patient‐Reported Outcomes and Late Toxicity After Postprostatectomy Intensity‐Modulated Radiation Therapy,” European Urology 76 (2019): 686–692. 31113644 10.1016/j.eururo.2019.05.011

[24] C. J. Wallis , P. Cheung , S. Herschorn , et al., “Complications Following Surgery With or Without Radiotherapy or Radiotherapy Alone for Prostate Cancer,” British Journal of Cancer 112 (2015): 977. 25688739 10.1038/bjc.2015.54 PMC4366895

[25] P. M. Arlen , F. Bianco , W. L. Dahut , et al., “Prostate Specific Antigen Working Group Guidelines on Prostate Specific Antigen Doubling Time,” Journal of Urology 179 (2008): 2181–2186. 18423743 10.1016/j.juro.2008.01.099 PMC2667701

[26] W. P. Fendler , J. Calais , M. Eiber , et al., “Assessment of 68Ga‐PSMA‐11 PET Accuracy in Localizing Recurrent Prostate Cancer: A Prospective Single‐Arm Clinical Trial,” JAMA Oncology 5 (2019): 856–863. 30920593 10.1001/jamaoncol.2019.0096 PMC6567829

[27] J. Calais , F. Ceci , M. Eiber , et al., “(18)F‐Fluciclovine PET‐CT and (68)Ga‐PSMA‐11 PET‐CT in Patients With Early Biochemical Recurrence After Prostatectomy: A Prospective, Single‐Centre, Single‐Arm, Comparative Imaging Trial,” Lancet Oncology 20 (2019): 1286–1294. 31375469 10.1016/S1470-2045(19)30415-2 PMC7469487

[28] V. Sharma , A. Nehra , M. Colicchia , et al., “Multiparametric Magnetic Resonance Imaging Is an Independent Predictor of Salvage Radiotherapy Outcomes After Radical Prostatectomy,” European Urology 73 (2018): 879–887. 29195777 10.1016/j.eururo.2017.11.012

