# Supplementary material for: Understanding the Impact of Salvage Radiation on the Long‐Term Natural History of Biochemically Recurrent Prostate Cancer After Radical Prostatectomy
Source: Cancer Med. 2025 Jun 26;14(13):e70988. doi: 10.1002/cam4.70988 (PMC12198693; doi:10.1002/cam4.70988)
Supplement: Supplementary file 1 — Data S1. Supporting Information. [file CAM4-14-e70988-s001.docx]

**Supplementary Methods**

**Mayo Clinic Prostatectomy Registry Follow Up Protocol**

Every patient who has undergone radical prostatectomy at Mayo Clinic is contacted annually to assess vital status, disease status (e.g., recurrence), and secondary treatment status. This follow-up is obtained by our registry abstractors and updated continuously. Indeed. We first send patients a mailed in survey inquiring about their post-operative PSAs and recurrence information indefinitely after surgery. The response rate on these survey’s is >95%. In addition, we also send surveys and correspondence to the patient’s local treating physicians (referring and primary care provider) to get more information about events (metastasis, salvage treatments, and death). Lastly, we also cross-reference our registry data with the national death index to confirm mortality and cause of death.

**Propensity Score Matching Details**

For each SRT-treated patient, we identified an untreated match, matching 1:1 using a greedy algorithm, as follows: first, for an SRT-treated patient who received SRT at *t* years post-BCR, we identified potential matches who were alive, event (metastasis)-free and without SRT at the equivalent time *t* years since BCR. Among these, the untreated match was selected based on the smallest Euclidean distance to the treated patient, using the propensity score hazard at *t* years and time since the most recent PSA. Patients treated with SRT were considered as a potential untreated match at earlier timepoints if their SRT was more than two years after the potential match timepoint. However, no SRT treated patients were selected as an untreated match. Data were randomly sorted before matching to optimize the greedy algorithm. Missing data for covariates were rare, and a complete case analysis was conducted.

**Supplementary Figure.** Cumulative incidence of systemic progression, stratified by receipt of SRT versus no SRT, subset to only patients with sufficient PSA doubling time information.

**
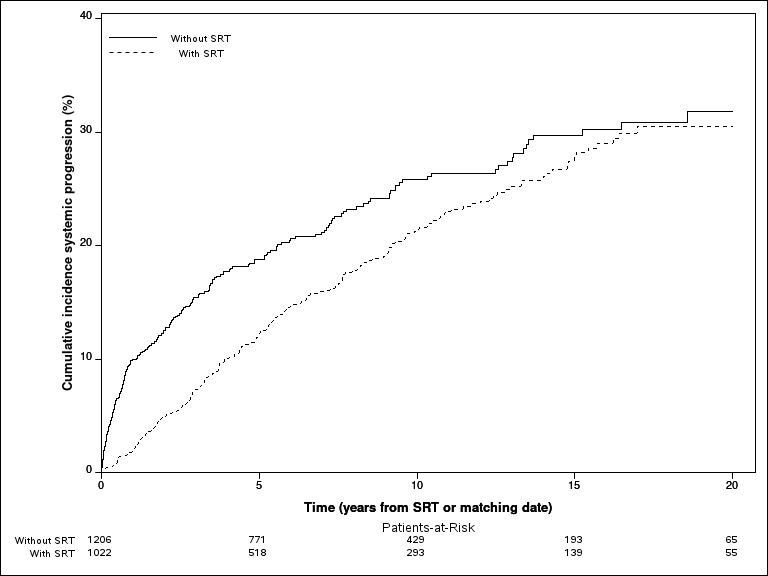
**

**P=0.0015**

**Supplementary Table.** Hazard ratios of SRT after matching by propensity scores, and Number Needed to Treat for systemic progression, prostate cancer specific mortality and overall mortality. Analyses subset to patients with PSA doubling time information and models adjusted for PSA doubling time.

| **Disease**  **Outcome** | **Hazard Ratio (95% CI)** | **P value** |
| --- | --- | --- |
|  |  |  |
| **Systemic progression** | 0.76 (0.59 – 0.98) | **0.031** |
| **Prostate cancer mortality** | 0.82  (0.57 – 1.17) | 0.274 |
| **Overall mortality** | 0.83  (0.70 – 1.00) | **0.0468** |
